# Effects of sensorimotor foot orthoses on static balance in healthy adults: a crossover randomized controlled trial

**DOI:** 10.3389/fspor.2025.1616696

**Published:** 2025-07-31

**Authors:** Stephan Becker, Steven Simon, Josefine Hayer, Jana Heidger, Wjatscheslaw Koltschin, Carlo Dindorf, Jonas Dully, Michael Fröhlich

**Affiliations:** Department of Sports Science, RPTU University of Kaiserslautern-Landau, Kaiserslautern, Germany

**Keywords:** SMFO, insoles, proprioception, center of pressure (COP), posturography, postural sway, fall prevention

## Abstract

**Background:**

Sensorimotor foot orthoses (SMFO) may be a helpful intervention to improve balance by enhancing proprioceptive input within the sensorimotor control loop. SMFO intervention could have beneficial effects on reducing risk of ankle sprains and falls.

**Methods:**

A total of 57 healthy adults (age: 48.5 ± 11.8 years) completed a static balance test (30 s) on a force plate, with open eyes (OE) and closed eyes (CE). Balance performance was assessed by using posturography to measure the sway area (mm^2^), under SMFO and no foot orthoses (NFO) conditions.

**Results:**

Descriptive statistics show a reduced median of 1.9 cm^2^ (29.6%) for SMFO with CE. No interaction was found, while main effects showed significant differences (vision: *p* < 0.001, sole: *p* = 0.004). *post-hoc* tests underlined these results and static balance improved by a median 0.8 cm^2^ (14.5%) with SMFO compared to NFO.

**Conclusion:**

The SMFO seems to support static balance and sensorimotor system, which could help to avoid falls and injuries as ankle sprains. Further age groups, long-term effects and the impact on dynamic balance must be studied.

## 1 Introduction

Balance is a fundamental ability, involved in almost all human movements and plays a central role in regulating of our posture (1). As a human being is not a rigid structure, gravitational forces and natural internal fluctuations, which can be generated by breathing, for example, must be compensated for by adequate muscle reactions while standing (2). Owing to the human bipedal stance and relatively high center of gravity, humans already have demanding prerequisites for maintaining balance (3).

Balance is also considered to be of great importance in injury prevention (4). A poor sense of balance can increase the risk of injury (5) and functional ankle instability (6). Older people are also more likely to fall because of poorer vestibular sense, among other factors (7). One explanation for the importance of balance with regard to injury prevention and fall prevention is that balance is controlled by the sensorimotor control circuit in addition to the vestibular organs (8). In addition to the visual and vestibular senses, the sensorimotor control circuit also includes various exteroceptors and proprioceptors that inform the CNS via afferent pathways regarding the sensory signals that are received, for example, via the sole of the foot or changes in muscle tension (4). The sum of the incoming feedback is processed subconsciously in the CNS, and the body reacts muscularly to achieve the desired state, by comparing the predicted and actual states (9, 10). The better the sense of balance, the faster the processing sensory information (4).

Balance training is a proven method to improve balance (1, 11) and reduce injuries (12) and functional ankle stability (13). However, not all people get to train regularly. Therefore, the potential added value of sensorimotor foot orthoses (SMFO) is increasingly coming into focus (14). The primary aim of SMFO is to change the foot kinetics and kinematics in a defined manner (tonus increase or tonus decrease) via specific elements at a certain point in the step cycle (15–17). The insole utilizes the exteroceptors and proprioceptors, which are stimulated by the SMFO elements. From the authors’ point of view, the combination of a toe bar and retrocapital could have a positive influence on balance. Combining both elements supposedly increases plantar flexor tension (15). The plantar flexors are the central control elements for static balance (7). In addition, the toe bar causes the toes to lay down, particularly if a person tends to curl their toes, which can increase the contact surface of the toes. With an increased contact area, the input of sensory information from exteroceptors in the skin could increase, which would be beneficial for balance (4, 14). SMFO has been used in practice for many years and was investigated for the first time in 2013 by a research group from Japan (18). Even though its use in practice has generated a lot of positive feedback, there is a need for evidence-based research into the postulated effect of the combination of the two elements.

To date, no studies have investigated the effects of SMFO on balance. However, some studies have investigated the influence of textured insoles on balance (19) and demonstrated that balance can be positively affected by changing afferent information from the foot sole (20).

Several validated methods exist for assessing balance, with computer-assisted posturography being a recognized static procedure, commonly referred to as the postural stability test (21, 22). Using a force plate, changes in the center of mass (COM) can be assessed using the center of pressure (COP), which is also observed in healthy individuals while standing completely still (23). A common issue seen in individual balance abilities, both static and dynamic, is the over-reliance on the visual sense (24–26). Without input from the visual receptors in the sensorimotor control circuit, many individuals struggle to attain similar balance measurement values (4, 27).

This study aimed to investigate the influence of SMFO on static balance in healthy adults. Using a randomized cross-over design, participants completed a posturographic balance measurement with SMFO and with no foot orthosis (NFO). We tested the following hypotheses:

• Wearing SMFO improves static balance during a 30 s measurement with open eyes (OE) and with closed (CE) compared to NFO.

The literature shows that, to date, there has been no study on balance with SMFO, which seems to be a promising intervention method that has been on the market for many years. Demographic changes, the growing number of elderly people, and the increasing social importance of injury prevention in general also support the value of such supportive measures. However, the necessary evidence must first be provided, which is why the first step should be to look at whether there are general effects of the SMFO on the balance in healthy people, regardless of a specific indication, being the next step.

## 2 Methods

### 2.1 Participants

A sample size was prior calculated using G*power (Version 3.1.9.6 for Macintosh, University of Kiel, Germany) for t-tests between two dependent means (matched pairs; effect size d = 0.5, 2 ToMs, α error probability = 0.05, power: 0.95). A minimum group size of 45 individuals was calculated. To account for potential dropouts, a total of 57 participants (sex: 28♀, 29 ♂; age: 48.5 ± 11.8 years; height: 1.73 ± 0.1 m; weight: 79.1 ± 15.2 kg; body mass index: 26.3 ± 4.0 kg/m^2^) were included in this crossover randomized controlled study. This study was conducted in accordance with the current guidelines of Declaration of Helsinki and was approved by the responsible ethics commission (No. 55). All participants provided written informed consent after receiving a full explanation of the study and agreeing to the participation and publication of the results.

The inclusion criterion was age >30 years, when postural balance starts declining (28, 29). The youngest participant was 30 years old, and the oldest participant was 68 years old. The exclusion criteria were diabetes mellitus, rheumatic diseases, and neurological disorders of the musculoskeletal system. Additionally, individuals with an acute foot or ankle injury in the past eight weeks were excluded. These exclusion criteria were selected because of their potential influence on balance. As this is the first study on SMFO and balance, a healthy sample was selected in order to check whether effects can already be seen here.

### 2.2 Foot orthoses

**Figure 1:**
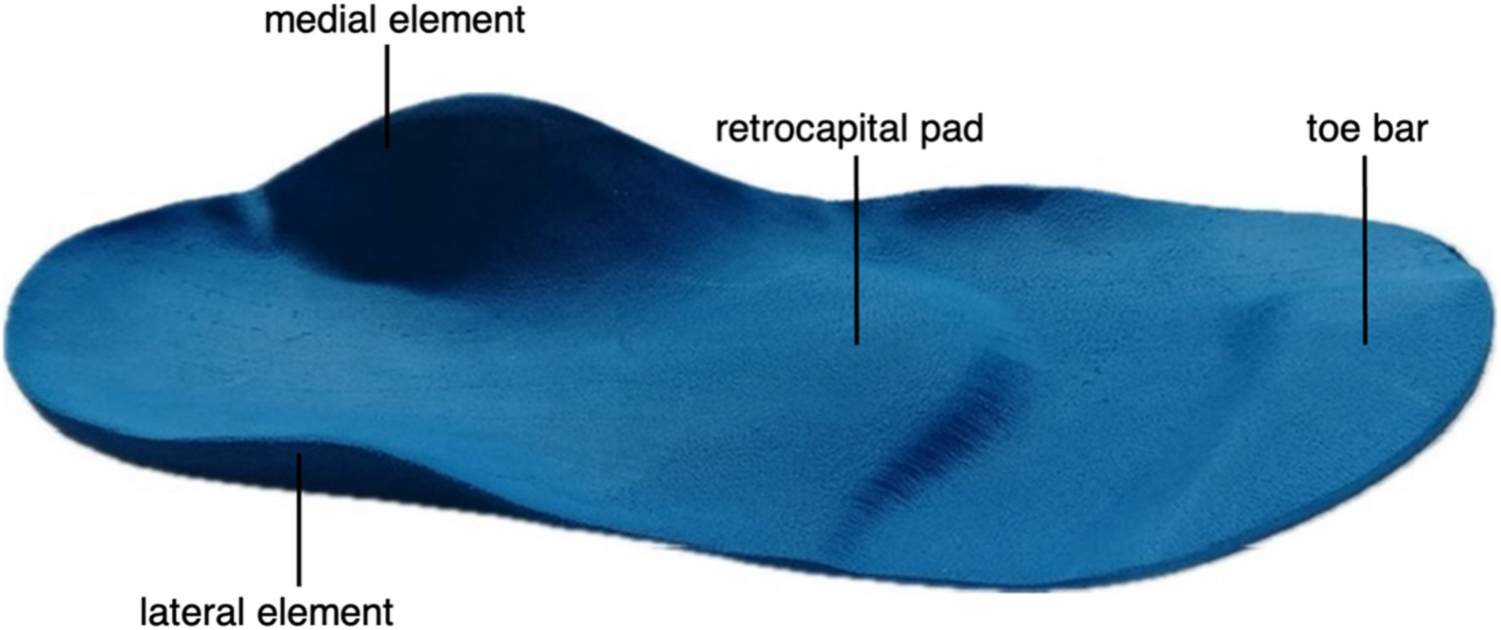
The SMFOs used in this study.

SMFOs were manufactured according to the Woltring/Springer and built using ethylene-vinyl acetate (EVA) material with a shore hardness of 35 by the same and experienced orthopedic shoe technician. The medial and lateral elements were grounded to prevent muscle activation or stabilization. In contrast, the toe bar (7.5–8.1 mm) and retrocapital pad (8.7–9.5 mm) were constructed in relation to the shoe size and not individually manufactured. The exact structure and the height ranges of the elements are given in Figure 1.

### 2.3 Procedure

**Figure 2:**
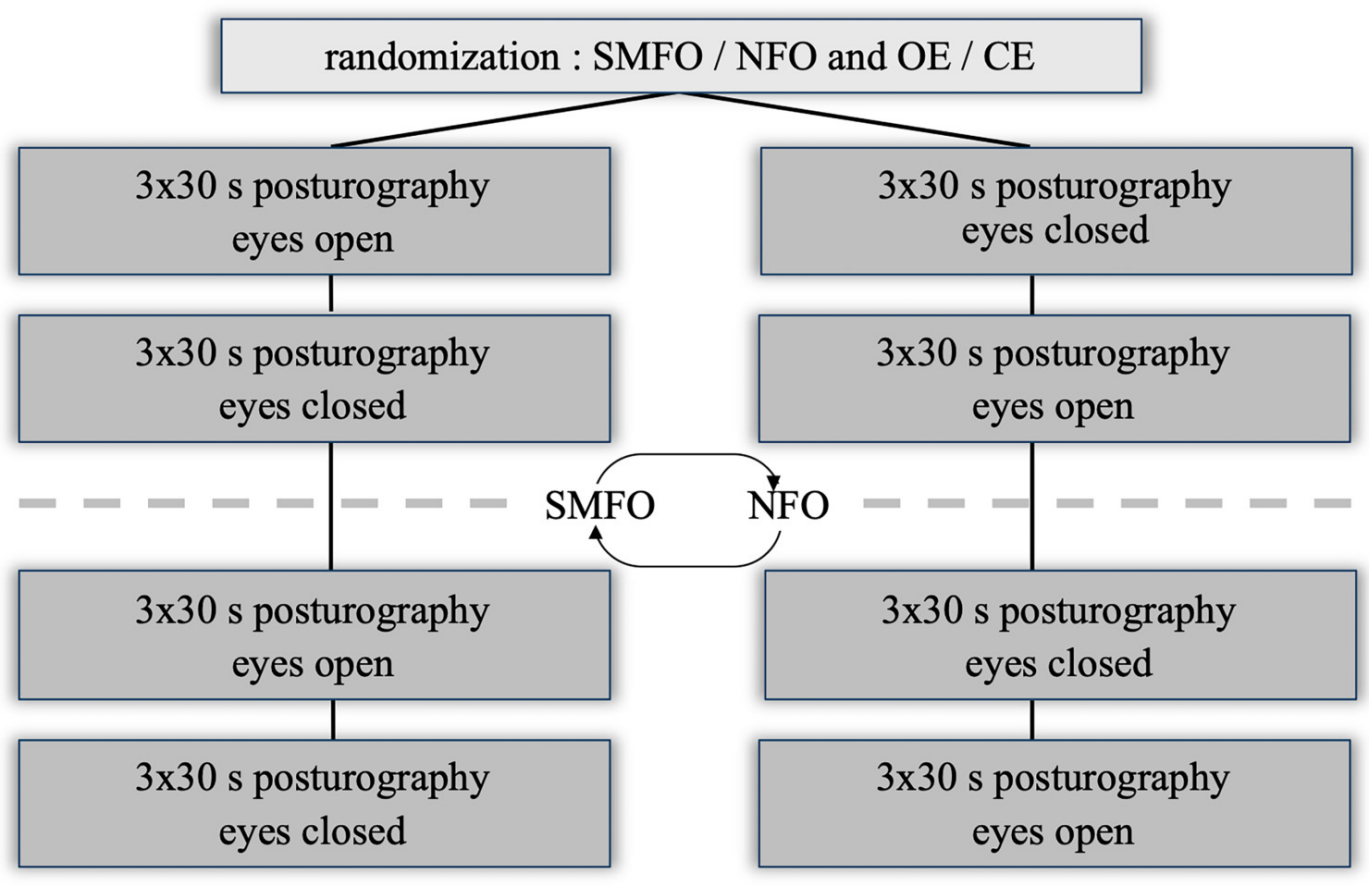
Study design. Test procedure (SMFO, sensorimotor foot orthoses; NFO, no foot orthoses; OE, open eyes; CE, closed eyes).

First, we randomly determined whether a person started an SMFO or NFO. Next, we randomly determined whether static balance was measured with OE or CE. Figure 2 illustrates the exact procedure followed after randomization. Measurements were conducted in the private setting for each participant using neutral shoes (model: Samba, Adidas AG, Herzogenaurach, Germany) with flat soles and without arch support. Static balance was analyzed using a posturographic measurement of 3 × 30 s (OE and CE) on a mobile force plate (model: GP Multisens, version: MS4, go-tec GmbH, Münster, Germany). The plate features 2.304 resistance sensors over a 39 × 39 cm surface (2 sensors per cm²) with a sampling rate of 200 Hz. Data processing and visualization were performed using the GP Manager (version 7, Go-tec GmbH, Münster, Germany) to derive the movement of the center of pressure, known as the area of the ellipse of the sway area (cm^2^). Out of the three tests performed for each of the conditions [SMFO (OE, CE); NFO (OE, CE)] mean value was used for the statistical analysis. The pressure plate was positioned facing the wall to reduce distraction. The measurements were performed without acoustic disturbances. The participants positioned themselves in a hip-width stance with the same stand width between the tests. The head was in an upright position, looking straight ahead, and the arms were relaxed on their sides. The participants were instructed to stand as still as possible and to visually fixate on a point on a wall during the tests with OE. The investigator and the display were outside of the field of view to ensure that there was no distraction and, in particular, no visual feedback on the movements of the COP.

### 2.4 Statistics

Statistical analyses were performed in R (version 4.4.2). The results are expressed as mean values ± standard deviations and 95% confidence intervals. Both, the orthoses (SMFO, NFO) and the setting (OE, CE) were seen as repeated measures factors. The various conditions were calculated in one model based common procedure in this discipline (30). Since the data violated normal distribution (31), a LD-F2 design with ANOVA-Type statistics was calculated using the R package nparLD (32) with effect sizes eta². Significant effects were *post-hoc* tested with a Wilcoxon signed-rank test with Pearson's r effect size. Statistical significance was set at *p* < 0.05. The outliers were evaluated by experts and found to be systematic. This was also confirmed by the methodological procedure, which always included the best of three attempts for all conditions in the statistics.

## 3 Results

**Figure 3:**
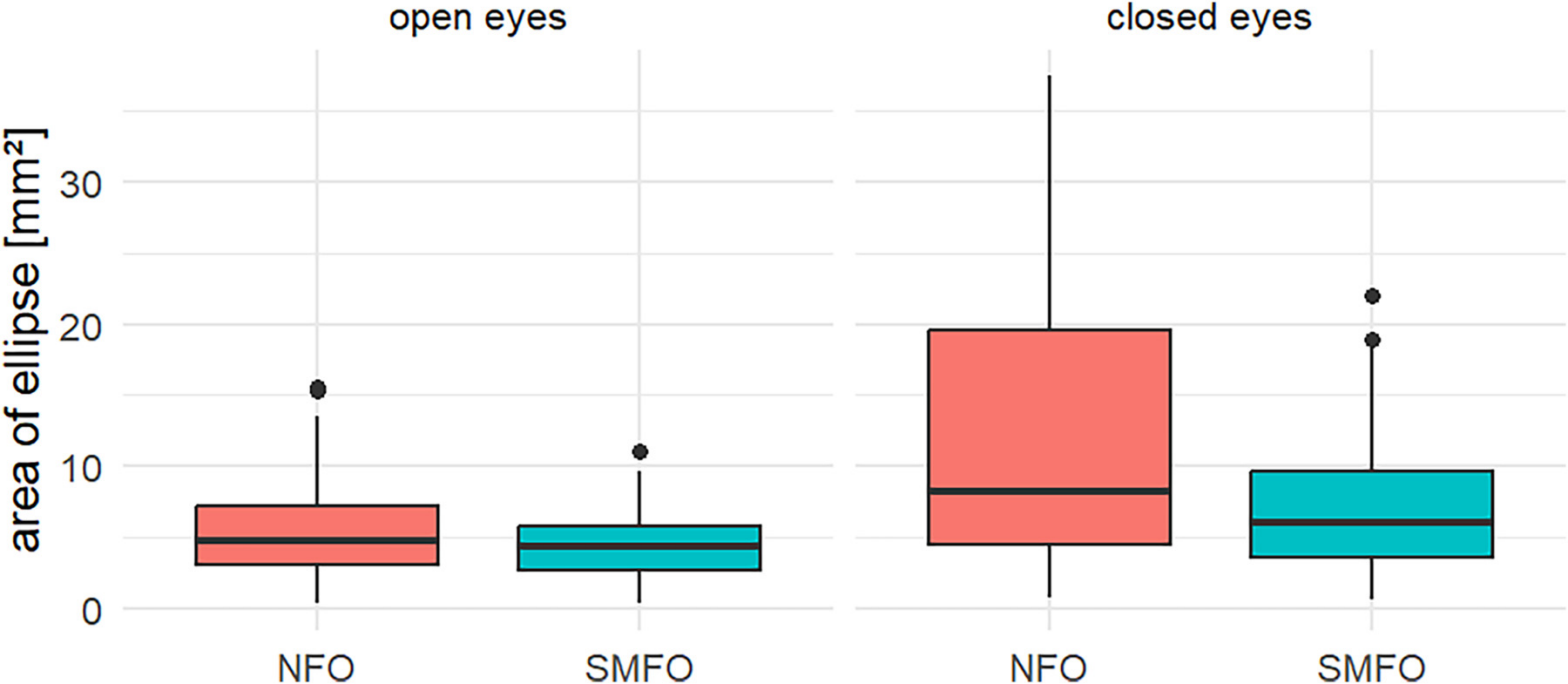
Boxplots representing descriptive statistics of both conditions (open eyes, closed eyes) for both type of soles (NFO, SMFO). Some of the outliers were removed to improve presentation with consistent scaling.

The descriptive statistics showed a reduction in the sway area while wearing SMFOs compared to NFO for OE and CE (Figure 3; Table 1).

**Table 1:** Descriptive data of the results (median, interquartile range).

Setting		SMFO			NFO		
	*n*	Median [cm^2^]	CV_robust_ [%]	IQR [cm^2^]	Median [cm^2^]	CV_robust_ [%]	IQR [cm^2^]
Open eyes	57	4.6	84.8	3.9	4.8	106.2	5.1
Closed eyes	57	6.4	139.0	8.9	8.3	209.6	17.4

The mean value for each participant and condition was calculated from three tests.

CV_robust_, robust coefficient of variation; IQR, interquartile range.

The results of the non-parametric ANOVA are presented in Table 2. Since sole (SMFO, NFO) and vision (OE, CE) both showed significant effects, Table 3 shows the *post-hoc* Wilcoxon tests for both effects.

**Table 2:** Results of the non-parametric ANOVA.

Effect	F-statistic	df	*p*-value	Eta²
Sole	7.94	1	0.004*	0.122Eta²
Vision	77.32	1	<0.001**	0.576Eta²
Interaction	0.29	1	0.5	0.005Eta²

*Significant on a level of *p* < 0.05.

**Significant on a level of *p* < 0.001.

**Table 3:** *Post-hoc*-Wilcoxon test for both main effects.

Effect	W-statistic	df	*p*-value	*d*
SMFO vs. NFO	4,163.5	56	<0.001**	0.63
OE vs. CE	6,098.5	56	<0.001**	2.27

SMFO, sensorimotor foot orthoses; NFO, no foot orthoses; OE, open eyes; CE, closed eyes.

**Significant on a level of *p* < 0.001.

## 4 Discussion

The results of the present study showed no interaction between the conditions sole and vision but two significant main effects (sole: *p* = 0.004, vision: *p* < 0.001). The significant difference for the vision is an additional result of the inferential statistics which can be expected and does not differentiate between SMFO vs. NFO and is therefore negligible. *post-hoc* tests underlined these results, where static balance improved by a median of 0.8 cm^2^ (14.5%) with SMFO compared to NFO (*p* < 0.001, d = 0.6).

Due to the lack of an interaction effect, no inference statistical differentiation is made between SMFO OE vs. NFO OE and SMFO CE and NFO CE. The descriptive statistics indicate that participants in particular benefited from the SMFO with CE (median: 1.9 cm^2^, 29.6%) compared to NFO.

The results support the hypothesis that the SMFO might be a helpful foot orthosis for improving balance regulation. This could be a helpful support for our sensorimotor system to avoid injuries or falls.

### 4.1 Possible neurophysiological explanations

From the authors’ perspective, there are several possible explanations for the positive influence of SMFO on balance regulation. The first explanation is based on a changed proprioception do to the fact that the combination and arrangement of the two elements (see Figure 1) causes the toe flexors to stretch, as the metatarsal heads fall into the gap between the toe bar and the retrocapital pad. Consequently, an increased muscle tone resolved via Ia afferents triggered by the muscle spindles (33). This indicated that the activation frequency of these muscular tension sensors increased in the afferent signal chain (33). The neuromuscular consequence is a contraction of the plantar foot muscles to protect them from further increases in stretch (33). In this case, however, as the participants were standing statically on the SMFO, it is probably only an isometric contraction and an increase in tension, as a concentric shortening of the muscle probably cannot be achieved mechanically due to the standing position. Nevertheless, an increase in the tension of the toe flexors may be beneficial for balance regulation. It is known that the toe flexors play an important role here (34), particularly while standing (35). In a further explanatory approach, it is conceivable that this increase in tension on the plantar aponeurosis could pull the calcaneus into a slight retroversion that transmits a similar supportive toning effect via the Ia afferents to the m. gastrocnemius and m. soleus (36), which also play an important role in balance regulation (37, 38).

The following explanation focuses on the toe bar and its primarily effects on mechanoception. This element of the SMFO is said to be beneficial for the pre-tension of the toe flexors and leads to a greater area of contact between toes and SMFO because of its shape. The increased contact surface can increase afferent information density via known mechanoceptors in the skin (20, 39), such as the Pacini corpuscles, Ruffini corpuscles, and Merkel cells. An increase in sensory information to the CNS seems to be a helpful factor, ashas been shown in other studies (19, 20). Furthermore, the toe bar might improve toe grip strength and standing balance, as Nakano et al. have already revealed for a slightly different toe grip bar (40).

Another explanation is the mechanical stimulus by the elements, which is altered by the surface profile compared to the NFO and causes new joint positioning, which could unconsciously have a greater impact on the afferent information from this area (41–43), which having a unspecific but somehow positive effect on balance regulation, in this case, COP control (19, 44).

The last explanation is a combination of the previous approaches. In conclusion, it should be emphasized that the results demonstrate the positive change with the present experimental design and methodology. Other studies are needed to answer this complex neurophysiological question and thus provide an evidence-based explanation. Nonetheless though the exact neurophysiological processes of the SMFO are unclear, this first study confirms the assumption that the SMFO positively influences balance.

### 4.2 Comparable investigations

To the best of our knowledge, this is the first study on SMFO and their effects on balance. Therefore, further studies must examine other forms of balance and participants. Nevertheless, previous studies have shown that balance can be influenced by the surface texture of the insole (19). These forms of insoles also influence the afferent information within the sensorimotor system. While the SMFO consists of four central elements, all of which have specific and targeted functions (toning and detoning of muscle tension) (15, 16) this form consists of flat, industrially manufactured insoles that only have a very small und unspecific textured surface that is the same at all points of the insole (19). However, based on the design in these cases, primarily via a change in mechanoception through the non-specific surface texture (spikes, pyramids and granulations) of the insole, there were no fundamental changes to the profile with specific and predefined objectives (19). Compared with other reference values for the area of the ellipse (45), the present values are higher on average, which is presumably due to the methodical approach: average value from three attempts, instead of the best value per condition (46).

Kalron, Pasitselsky (47) showed that in 25 patients with MS with an insole containing miniature square pyramids, CE led to immediate improvements in static balance (sway path length and sway rate). A significant observation was also confirmed after a wearing period of 4 weeks. As in the present study, Kalron, Pasitselsky (47) did not find any significant improvements with OE. Hatton, Dixon (48) made similar observations in 50 healthy adults. With OE, static balance (medio-lateral sway) improved significantly, by 9.2% when wearing an insole with pyramidal peaks. The same authors also found a significant improvement in balance (sway velocity) in patients with diabetic neuropathy (49). A textured insole with a nodule design was used, whereby the intervention group with the textured insole improved significantly, by 5%, after a wearing duration of 4 weeks in the static test with OE. Qiu, Cole (50) showed that patients with Parkinson disease significantly improved their static balance (medio-lateral sway) by wearing textured insoles with granulations across the upper surface. Other studies with a similar designs also demonstrated the positive influence of textured insoles with significant results, particularly with CE (51).

All of them are only partially comparable with the present study, as these types of mainly industrially manufactured insoles must be distinguished from the SMFO by their variation of specific manufactured elements. The insoles used in these studies primarily work with an unspecific and consistently structured surface texture, whereas the SMFO uses different elements that lead to mechanical position changes in the feet and lower extremities via a specific profile with the respective objectives (18, 52–54). Treatment with an SMFO is usually based on a specific indication that is also known from biomechanical foot orthosis treatment and is an individual, handcrafted product. For SMFO, the improvement in balance can therefore, be described as a positive side effect, which can make sense in several clinical cases that affect balance regulation, such as multiple sclerosis (47, 55), Parkinson disease (56), or polyneuropathy (49). However, a comparison with the studies presented shows that balance can be influenced by the sole of the foot and the afferent information to the CNS (20). Furthermore, the findings show that even if the heterogeneity of the collected parameters and participants weakens the comparability, that people seem to benefit from the insoles, especially when their eyes are closed.

Nevertheless, some studies have not shown significant improvements in static balance (57–59). However, it is important to note that, thus far, there have not been any opposing trends that would worsen the balance via these approaches (19).

### 4.3 Relevance

Although the connection between SMFO and balance requires further confirmatory studies, this topic is highly relevant in practice. Demographic changes and the resulting increase in the proportion of older people are some of the reasons for this. The sense of balance declines continuously from middle age onwards (60, 61). This contributes to an increased number of falls among the elderly population (7). In this population group, the declining visual sense also plays a role (62), so that SMFO could presumably be particularly helpful for these people, as the results with CE show. Consequently, SMFOs could support fall prevention in the elderly population, even though improved balance has a positive side effect in the majority of SMFO treatments. The actual reason for SMFO treatment will probably be a specific indication [e.g., pes planovalgus (63)] or corresponding kinematic abnormalities that justify preventive action.

Furthermore, SMFO may be more important in the context of rehabilitation. Owing to its muscle-activating properties (63, 64), it may be of increased interest to clients anyway. Proprioceptive training, which is basically a type of balance training, is also an essential component of many post-traumatic or post-operative rehabilitation programs, as proprioceptive abilities have a stabilizing and therefore protective effect on joint kinematics (4, 12). This training effect might be enhanced by using SMFO.

Since the ability to maintain balance is often functionally limited because the sensorimotor status is deficient (8), SMFO could make a positive contribution to minimizing the risk of functional ankle instability, as the static balance is considered a predictor for ankle injuries (6). Especially if the SMFO is equipped with the lateral and medial spot in an individually adapted form. Ludwig and colleagues (64) have already been able to demonstrate that the lateral spot of a SMFO significantly increases the muscle activity of the m. peroneus longus. As a main foot pronator, the m. peroneus plays an important role in securing the ankle joint against supination strains. Ludwig and colleagues showed that both the integrated electromyographic output increased and that earlier activation of the same muscle was initiated. No lateral and medial spot were used for this study, as the focus was on the two anterior elements (toe bar and retrocapital pad). Nevertheless, the toe bar and the retrocapital pad already appear to improve the sensorimotor status and improving the static balance, which could be beneficial for the ankle stability.

Although there are evidence-based positive correlations between balance and, for example, the likelihood of injury (5, 6, 65), we must await further research to better assess the clinical relevance of a improved sway area for everyday life. Similarly, further research is needed to explicitly assess the difference between OE and CE of the different conditions (e.g., SMFO OE vs. NFO OE). This step was not taken due to the lack of an interaction effect (Table 2). The general difference between OE and CE, as applied here due to the statistical procedure used, is negligible from the authors’ point of view. The primary argument is the difference that the visual sense as one of the most important supporters of the sense of balance is absent (26). Nevertheless, both parts of the study are important, as they reflect everyday life on the one hand (OE) and reduce the focus on internal sensorimotor control circuits on the other (CE) (45).

### 4.4 Limitations

Despite these positive results, there are some limitations that restrict their generalizability. This is the first study on balance in combination with SMFO. The sample size was appropriate and healthy, but the results cannot be applied to younger people or older people, or to people with specific diseases. It is questionable whether SMFO would be useful for younger people, as their sense of balance might still be sufficiently developed. Balance has several dimensions. Whether these effects can be positively transferred to a dynamic balance needs to be investigated. Furthermore, this study analyzed only short-term effects, and no statements about the long-term effects can be made. Moreover, to calculate the group differences, the mean values of three measurements per condition were determined. This step results in a high coefficient of variation for all groups (46), which should be noted for future studies and interpretation. Limiting yourself to the respective best value could also be a suitable approach. Posturography is often only used with one measurement per condition in practical screenings.

It should be noted that the SMFOs in this study were prefabricated for all shoe sizes by an experienced master craftsman. This means that the retrocapital pads and the toe bars were adjusted to an average shape in relation to the foot size. However, they were not individually manufactured for each participant. However, from the authors’ point of view, however, this should be seen as a positive rather than a negative aspect, as an individual fitting should be more precise.

Follow-up studies should also compare BMFO as a control variable or target the influence of fatigue (66) as a confounding variable in combination with SMFO. It is conceivable that in the case of intentional neuromuscular fatigue, the support of the SMFO could lead to even better effects. Furthermore future studies should add self-reported ankle stability questionnaires to combine the objective results with the subjective perception.

## 5 Conclusion

SMFO may be a helpful intervention for improving static balance (sway area) and supporting our sensorimotor system to avoid injuries (e.g., ankle sprains) or falls. Further research is needed to determine the extent to which the combination of a retrocapital pad and a toe bar effectively supports different dimensions of balance, its long-term effects and clinical relevance. Also, to include natural variation and therefore a more ecologically valid measure, future investigations should consider analyzing more trials per condition.

## Data Availability

The raw data supporting the conclusions of this article will be made available by the authors, without undue reservation.
